# Comparison of the Antimicrobial Efficacy of Conventional Versus Chitosan Re-inforced Heat-Polymerized Polymethylmethacrylate Dental Material: An In Vitro Study

**DOI:** 10.7759/cureus.68856

**Published:** 2024-09-07

**Authors:** Thirupugaz Ramamurthy, Shafath Ahmed, Vidyashree V Nandini, Shiney Boruah

**Affiliations:** 1 Prosthodontics, SRM Kattankulathur Dental College and Hospital, Chennai, IND

**Keywords:** antimicrobial efficacy, c.albicans, candida albicans, chitosan, heat polymerised polymethylmethacrylate, streptococcus mutans

## Abstract

Introduction: Polymethylmethacrylate (PMMA) is widely used in the fabrication of dentures due to its aesthetic appeal and mechanical strength. However, PMMA's susceptibility to microbial colonization often leads to oral infections such as denture stomatitis. Enhancing the antimicrobial properties of denture materials is crucial for improving patient outcomes. Chitosan, a natural biopolymer, possesses inherent antimicrobial properties and could potentially enhance the microbial resistance of PMMA. This study has investigated the potential of chitosan-reinforced heat-polymerized PMMA denture material to reduce microbial colonization.

Aim: The aim of the study was to evaluate and assess the anti-bacterial and antifungal properties of chitosan-reinforced heat-polymerized PMMA with conventional heat-polymerized PMMA

Materials and methods: Chitosan-reinforced PMMA samples were fabricated with varying chitosan concentrations (0% control, 5%, 10%, and 15% by weight). The fabrication involved mixing chitosan powder with PMMA powder, adding monomer liquid, followed by mixing, packing, and curing using the conventional heat polymerization technique. The antimicrobial efficacy was assessed in vitro using two common oral pathogens: *Streptococcus mutans* and *Candida albicans*. Blood agar plates were used for *S. mutans* and Sabouraud agar plates were used for *C. albicans*. Each sample was placed on the respective agar plates inoculated with a standardized microbial suspension and incubated at 37°C for 24 hours. The number of colony-forming units (CFUs) was counted to quantify microbial growth. Statistical analyses, including linear regression analysis, one-way ANOVA test, and Pearson correlation were performed to evaluate the relationship between chitosan concentration and antimicrobial efficacy. The p-value was calculated to determine the statistical significance of the results.

Results: The chitosan-reinforced PMMA samples showed significantly greater antimicrobial efficacy compared to the conventional PMMA samples. The CFU counts for both *S. mutans* and *C. albicans* decreased with increasing chitosan concentration. Linear regression analysis indicated a strong negative correlation between chitosan concentration and CFU counts, with Pearson correlation coefficients of -0.97 for *S. mutans* and -0.98 for *C. albicans*. ANOVA analysis revealed a statistically significant difference in antimicrobial efficacy across different chitosan concentrations (p < 0.001).

Conclusion: Incorporating chitosan into heat-polymerized PMMA significantly enhances its antimicrobial properties against *S. mutans* and *C. albicans*. The antimicrobial efficacy improves with higher concentrations of chitosan, with the 15% chitosan-reinforced samples showing the most substantial reduction in microbial growth. These results suggest that chitosan-reinforced PMMA dentures could be a superior alternative to conventional PMMA dentures, potentially reducing denture-related infections and improving oral health outcomes for denture wearers.

## Introduction

Polymethylmethacrylate (PMMA) has been the material of choice for denture bases due to its favorable aesthetic properties, ease of processing, and adequate mechanical strength. However, one significant drawback of PMMA dentures is their susceptibility to microbial colonization, leading to denture stomatitis [1]. This microbial growth not only affects the longevity of the denture but also has significant implications for oral and systemic health [2]. As such, enhancing the antimicrobial properties of PMMA dentures has become a critical focus in dental materials research.

Chitosan, a natural biopolymer derived from chitin, has garnered considerable attention due to its inherent antimicrobial properties, biocompatibility, and biodegradability [3]. The integration of chitosan into dental materials has been explored as a potential method to combat microbial adhesion and proliferation on denture surfaces [4]. Chitosan's cationic nature allows it to interact with negatively charged microbial cell membranes, resulting in the disruption of the cell membrane and subsequent microbial death [5]. Thus, chitosan-reinforced PMMA (Ch-PMMA) has emerged as a promising material for the development of antimicrobial denture bases.

Previous research has demonstrated that chitosan incorporation can enhance the antimicrobial properties of PMMA [6], but comprehensive comparative studies specifically focusing on its efficacy against a wide range of oral pathogens are limited [7]. Such studies are essential to substantiate the potential benefits of Ch-PMMA dentures over conventional PMMA dentures and to understand the underlying mechanisms driving these antimicrobial effects.

This study aims to compare the antimicrobial efficacy of Ch-PMMA dentures with conventional PMMA dentures through in vitro experimentation. By systematically evaluating microbial adhesion and growth on these two types of dentures, we seek to determine whether the incorporation of chitosan can provide a significant antimicrobial advantage. The findings from this research could pave the way for the development of more effective, long-lasting denture materials that reduce the incidence of denture-related infections and improve oral health outcomes for denture wearers.

## Materials and methods

This was an in vitro, prospective study conducted in the Department of Prosthodontics, Crown and Bridge, SRM Kattankulathur Dental College and Hospital and Department of Microbiology, SRM Medical College Hospital and Research Institute, Chennai, Tamil Nadu India. The study was approved by the Institutional Ethical Committee SRM Institute of Science & Technology (Deemed to be University) dated August 11, 2023 (approval number: SRMIEC-ST0723-1406).

Sample size calculation

The sample size was calculated using G*Power Statistics (Heinrich-Heine-Universität Düsseldorf, Düsseldorf, Germany). The minimum sample size required was estimated to be 24. Hence, each group consisted of six samples.

Preparation of the sample

The low molecular weight chitosan powder (Molecular Weight: 50,000 Da, Product No: 448869; Sigma-Aldrich, St. Louis, Missouri, United States) was synthesized with the heat-polymerized acrylic resin in four varying percentages. The varying percentages were measured with the aid of an electronic balance scale. The specimens under examination were divided into four groups, namely: Ch0 (0%, control), Ch5 (5%), Ch10 (10%), and Ch15 (15%). Ch0 was the prescribed control group with 0% chitosan by weight. The measured and weighed chitosan particles were amalgamated with the heat-polymerized acrylic using the conventional technique. The manufacturer’s powder and liquid proportion was followed for heat-polymerized acrylic resin.

**Figure 1:**
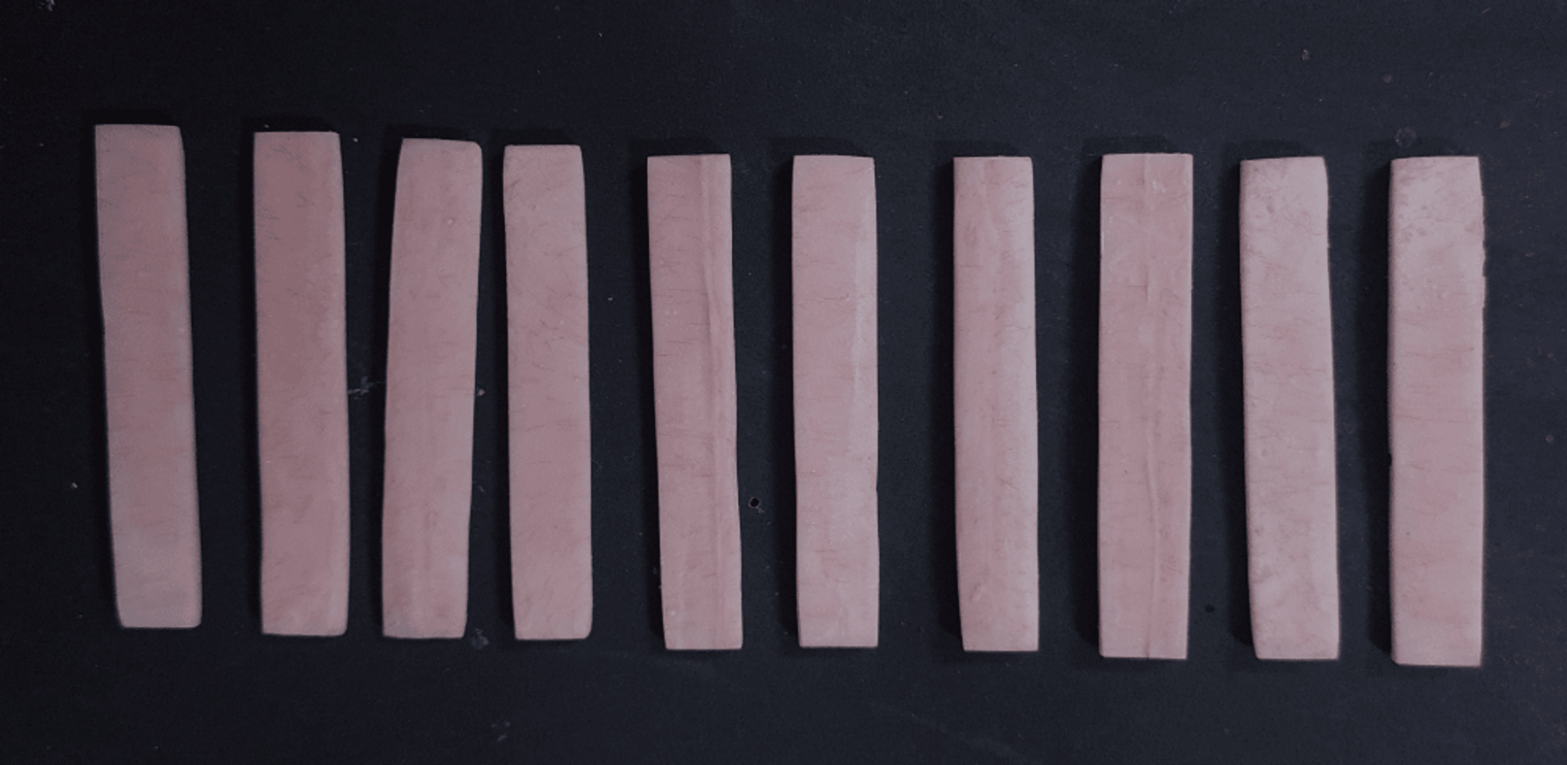
Prepared samples of chitosan-reinforced polymethylmethacrylate in varying percentages (0%, 5%, 10%, and 15%)

The wax patterns measuring 64 x 10 x 2.5 mm were fabricated, flasked, dewaxed, packed with the above-prepared combination, and polymerized by a conventional heat-polymerization cycle. The completely polymerized samples were surface trimmed with acrylic stones and underwent the application of grits of sandpaper and pumice polishing. The dimensions of the above-prepared samples were counter-checked with the aid of a digital vernier caliper with an accuracy of 0.01 mm (Figure 1) [8]. The processed and measured samples were stored in distilled water inoculated with *Streptococcus mutans* and *Candida albicans* at a temperature of 38±2°C with normal atmospheric pressure for 48 hours before sampling.

Bacterial assay

**Figure 2:**
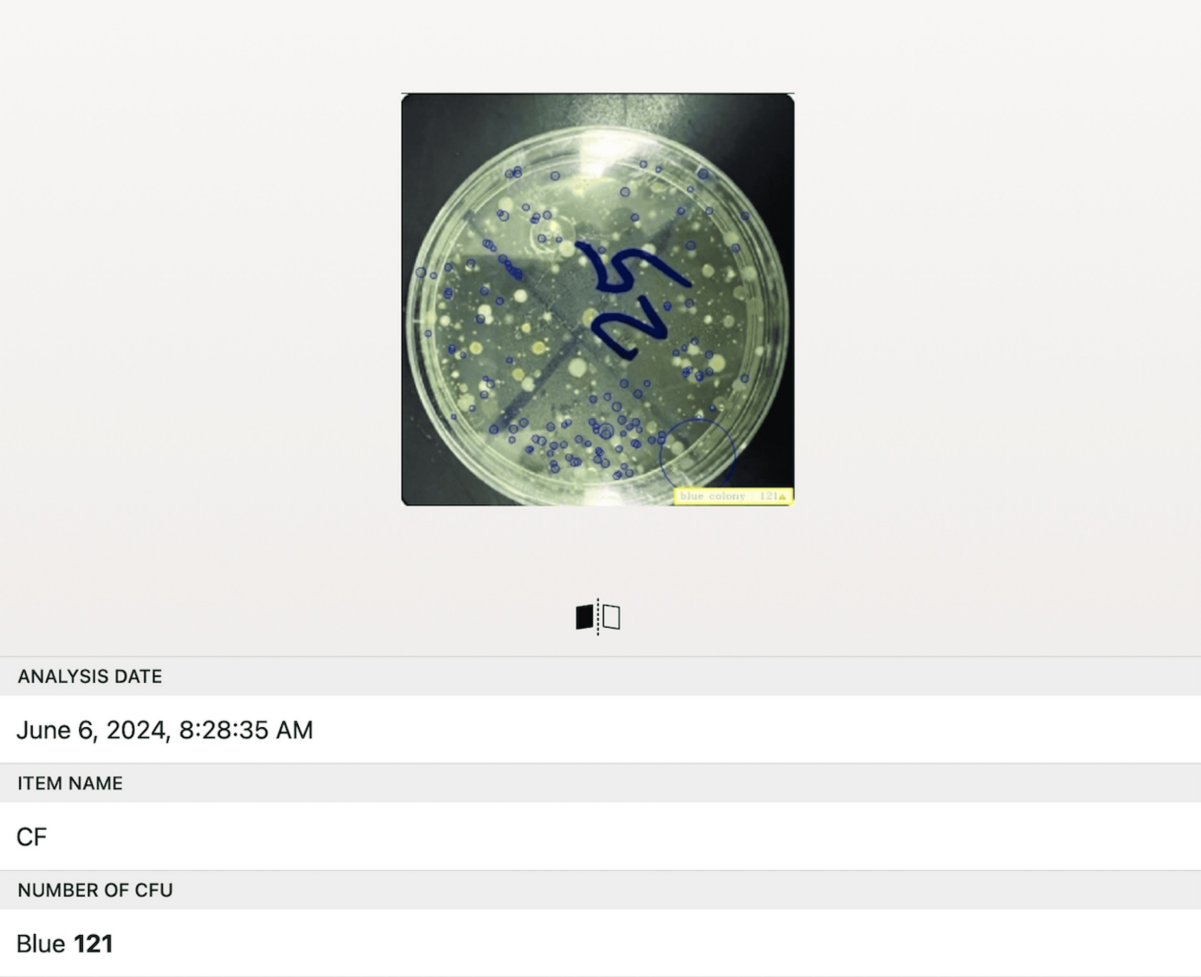
A screen of the automated image analysis software (@BactLAB™) used for counting the number of colonies of Streptococcus mutans and Candida albicans formed

The stored samples were assessed for antimicrobial efficacy and activity with the aid of colonization of *S. mutans* and *C. albicans*, which are the most commonly encountered microbial flora in the oral biome. The samples were inoculated and isolated on blood agar and Sabaroud agar plates for the isolation of *S. mutans* and *C. albicans,* respectively, for 24 hours at 37°C [9,10]. The number of colonies formed on the culture plates was counted through automated counting digital image analysis software (@BactLAB™) (Figure 2) [11]. The growth of the micro-organisms was assessed by establishing the colony-forming unit (CFU)/ml with the following formula [12]: 

 CFU/ml = Number of Colonies x Dilution Factor/Volume of Inoculum (mL) 

Statistical analysis

The various measurements and data corresponding to the antimicrobial activity of Ch-PMMA in various concentrations against *S. mutans* and *C. albicans* were recorded. Descriptive statistics were calculated. The mean values for each of the study groups (Ch0, Ch5, Ch10, Ch15) were derived. Statistical analyses, including linear regression analysis, one-way ANOVA test, and Pearson correlation were performed to evaluate the relationship between chitosan concentration and antimicrobial efficacy. The p-value was calculated to determine the statistical significance of the results. The computation for all the statistical analysis was performed using IBM SPSS Statistics for Windows, Version 27.0 (Released 2020; IBM Corp., Armonk, New York, United States). 

## Results

Growth of *S. mutans* in blood agar plates from prepared Ch-PMMA samples

**Figure 3:**
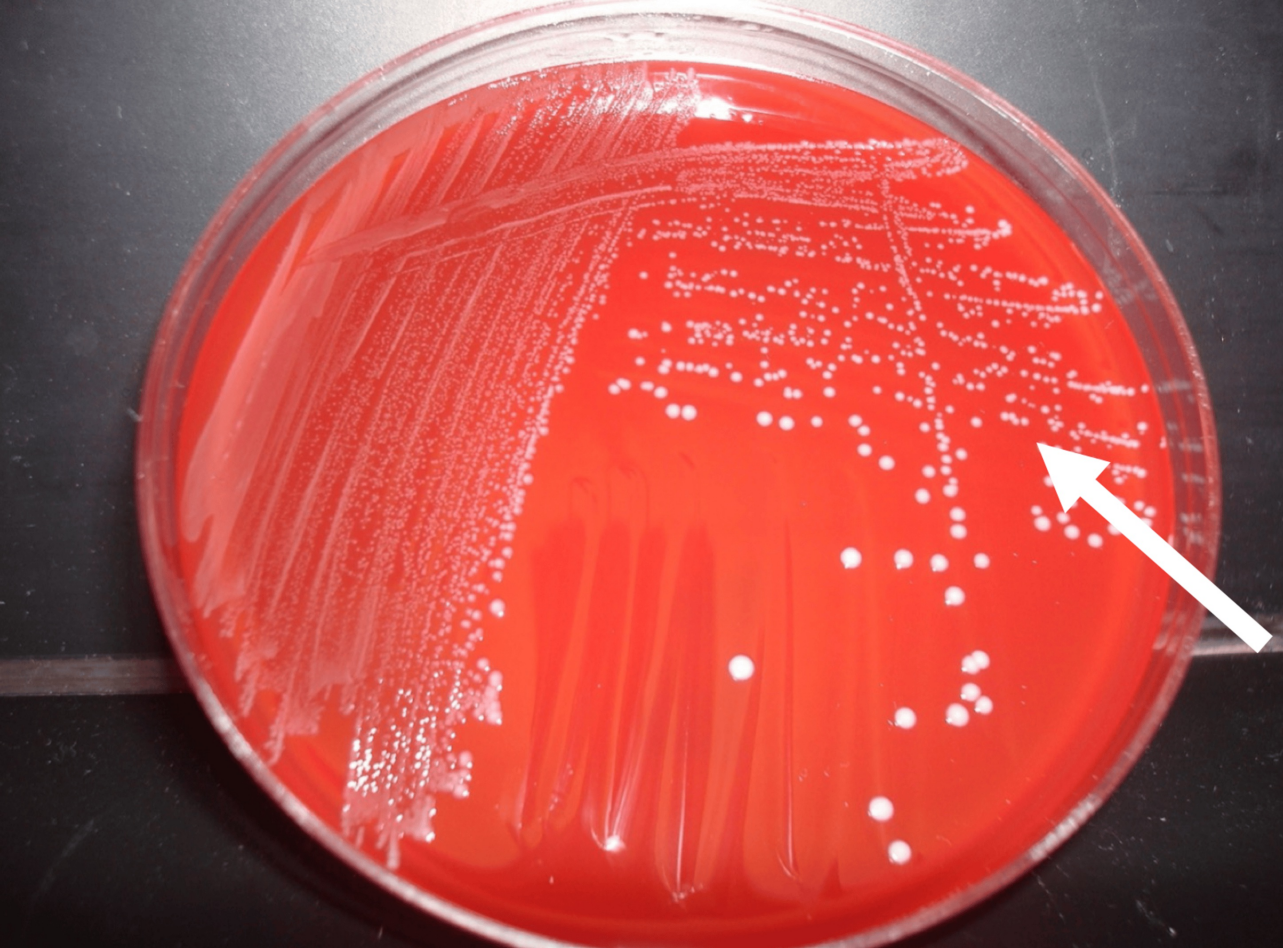
Microbial growth of Streptococcus mutans in blood agar plate from 15% chitosan-reinforced polymethylmethacrylate sample exhibiting least growth

The antimicrobial activity against *S. mutans* of the prepared Ch-PMMA samples of varying percentages (0%, 5%, 10%, and 15% chitosan by weight) was highly enhanced in the 15% Ch-PMMA samples with the least microbial growth (Table 1, Figure 3). Linear regression analysis indicated a strong negative correlation between chitosan concentration and CFU counts, with Pearson correlation coefficient, r = -0.97. ANOVA analysis revealed a statistically significant difference in antimicrobial efficacy across different chitosan concentrations (p < 0.001). 

**Table 1:** Growth of Streptococcus mutans in blood agar plates from the prepared Ch-PMMA samples of 0%, 5%, 10%, 15% Pearson's correlation coefficient (r) = -0.97, p-value = p < 0.001 Ch-PMMA: chitosan-reinforced polymethylmethacrylate

S.No	Sample Group (% of chitosan)	Growth of Streptococcus mutans in Blood Agar Plate
1.	Ch0 (0%, Control)	320 CFU/ml
2.	Ch5 (5%)	188 CFU/ml
3.	Ch10 (10%)	92 CFU/ml
4.	Ch15 (15%)	48 CFU/ml

Growth of C. albicans in Sabouraud agar plates from prepared Ch-PMMA samples

**Figure 4:**
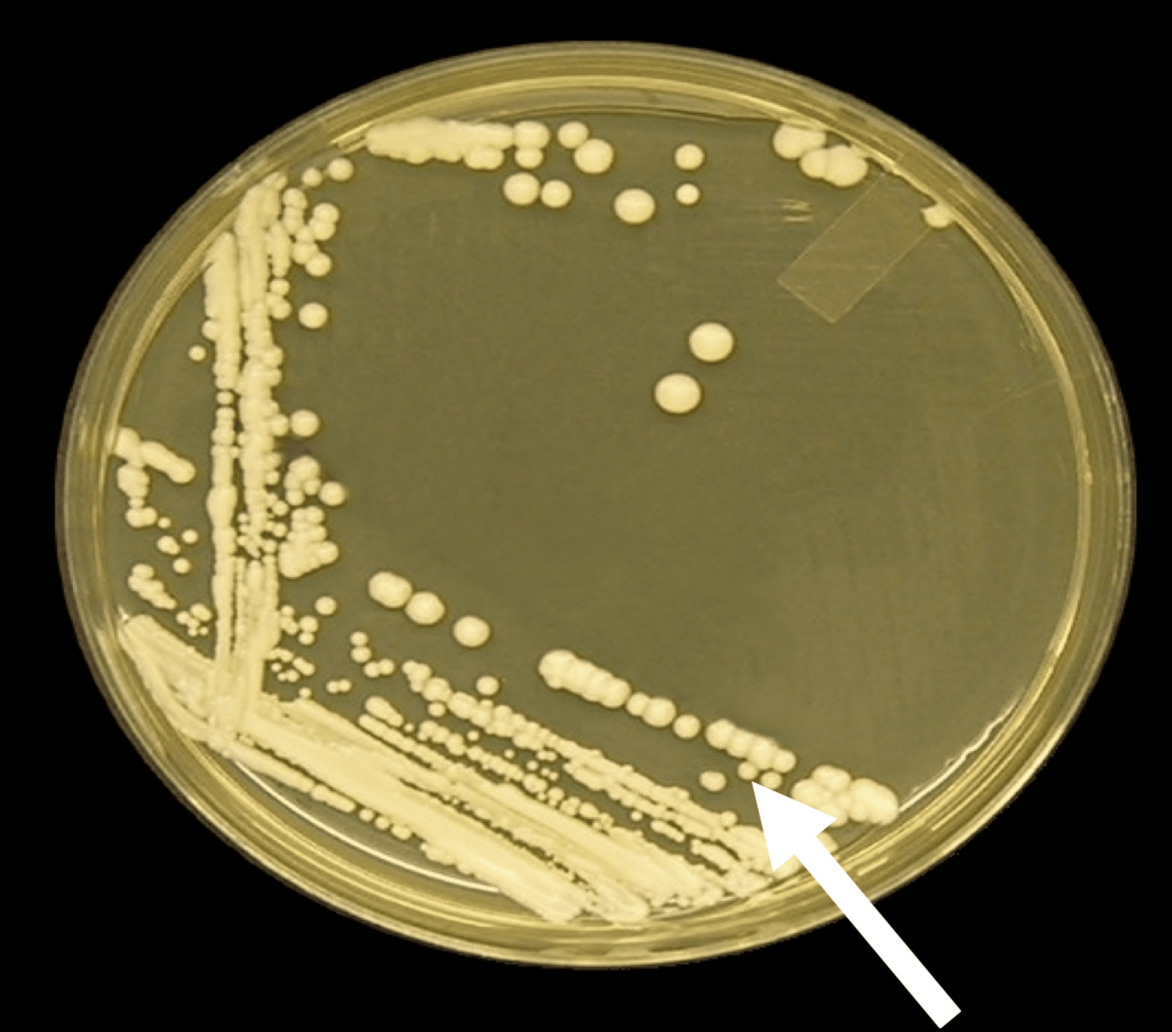
Microbial growth of Candida albicans in Sabouraud agar plate from 15% chitosan-reinforced polymethylmethacrylate sample exhibiting least growth

The antimicrobial activity against C. albicans of the prepared Ch-PMMA samples of varying percentages (0%, 5%, 10%, and 15% chitosan by weight) was highly enhanced in the 15% Ch-PMMA samples with the least microbial growth (Table 2, Figure 4). Linear regression analysis indicated a strong negative correlation between chitosan concentration and CFU counts, with Pearson correlation coefficient, r = -0.98. ANOVA analysis revealed a statistically significant difference in antimicrobial efficacy across different chitosan concentrations (p < 0.001). 

**Table 2:** Growth of Candida albicans in Sabouraud agar plates from the prepared Ch-PMMA samples of 0%, 5%, 10%, 15% Pearson's correlation coefficient (r) = -0.98, p-value = p < 0.001 Ch-PMMA: chitosan-reinforced polymethylmethacrylate

S.No	Sample Group (% of chitosan)	Growth of Candida albicans in Sabouraud Agar Plate
1.	Ch0 (0%, Control)	286 CFU/ml
2.	Ch5 (5%)	178 CFU/ml
3.	Ch10 (10%)	78 CFU/ml
4.	Ch15 (15%)	36 CFU/ml

## Discussion

Growth of *S. mutans* in blood agar plates from prepared Ch-PMMA samples

The results of this study demonstrate a significant reduction in microbial growth of *S. mutans* on blood agar plates with increasing percentages of chitosan reinforcement in the PMMA material. Specifically, the ch-PMMA samples exhibited a clear dose-dependent antimicrobial effect, with the Ch15 group (15% by weight of chitosan) showing the highest antimicrobial potency. This trend aligns with previous findings in the literature, further substantiating the efficacy of chitosan as an antimicrobial agent in dental materials.

Several studies have highlighted the antimicrobial properties of chitosan. For instance, Morsi et al. reported that chitosan incorporation into dental materials significantly inhibited the growth of common oral pathogens, including *S. mutans*, by disrupting microbial cell membranes and biofilm formation [4]. Similarly, Piras et al. found that chitosan-treated surfaces demonstrated a marked reduction in bacterial adherence and biofilm viability [13]. Our findings corroborate these studies, indicating that higher concentrations of chitosan in PMMA not only reduce microbial growth but also enhance the material's overall antimicrobial efficacy in comparison to the conventional PMMA denture base material which exhibits significantly lower antimicrobial properties. Furthermore, the study by Campoccia et al. provided evidence that chitosan's antimicrobial effect is primarily due to its ability to disrupt the cell walls of gram-positive bacteria like *S. mutans* through its cationic properties [5].

The present study's observation that the Ch15 group exhibited the highest reduction in microbial growth supports this mechanism, suggesting that the increased chitosan content amplifies its cell wall-disruptive action. Compared to conventional PMMA dentures, which exhibited no inhibition of microbial growth, the Ch-PMMA samples demonstrated substantial antimicrobial activity. This aligns with the findings of Giti et al. who reported that modifying PMMA with antimicrobial agents could significantly reduce microbial colonization and enhance denture hygiene [7]. Our study extends this understanding by quantitatively showing that the degree of microbial inhibition correlates with the percentage of chitosan added, thus providing a clear guideline for optimizing the antimicrobial properties of denture materials.

Growth of *C. albicans* in Sabaroud agar plates from prepared Ch-PMMA samples

The results of this study demonstrate a significant reduction in microbial growth of *C. albicans* on Sabouraud agar plates with increasing percentages of chitosan reinforcement in the PMMA material. Specifically, the Ch-PMMA samples exhibited a dose-dependent antimicrobial effect, with the Ch15 group (15% by weight of chitosan) showing the highest antimicrobial potency. These findings are consistent with previous research on the antimicrobial properties of chitosan in dental materials, reinforcing its potential application for improving denture hygiene. Several studies have investigated the antifungal properties of chitosan. For instance, Shih et al. reported that chitosan incorporation into dental materials significantly inhibited the growth of C. albicans by interfering with cell membrane integrity and disrupting cellular function [14]. Our results align with these findings, demonstrating that higher concentrations of chitosan in PMMA result in enhanced antifungal activity, as evidenced by the substantial reduction in *C. albicans* growth in the Ch15 samples. Moreover, a study by Aranaz et al.. found that chitosan exhibits strong antifungal properties due to its ability to bind to fungal cell walls, leading to increased cell permeability and eventual cell death [15]. This mechanism supports our observations, where the increased chitosan content in the Ch15 group correlates with the highest inhibition of* C. albicans *growth. The enhanced antimicrobial efficacy observed in this study suggests that chitosan effectively disrupts the fungal cell wall, thereby inhibiting growth and proliferation. In comparison to conventional PMMA dentures, which showed no inhibition of *C. albicans* growth, the Ch-PMMA samples demonstrated significant antifungal activity. These results are consistent with the findings of Maric et al., who reported that modifying PMMA with antimicrobial agents can significantly reduce microbial colonization, including that of fungal species like *C. albicans* [16].

Our study extends this understanding by quantitatively showing that the degree of fungal inhibition is directly proportional to the percentage of chitosan incorporated, providing a clear guideline for optimizing antifungal properties in denture materials. Additionally, similar findings were reported by Hussain et al., who observed that chitosan-modified dental materials exhibited improved resistance to fungal biofilm formation, thereby enhancing the longevity and hygiene of dental prostheses [17]. The significant reduction in *C. albicans* growth observed in our study, particularly in the Ch15 group, underscores the potential of Ch-PMMA as a superior alternative to conventional PMMA for denture bases, offering enhanced protection against fungal infections [18].

Limitations of the study

This study's in vitro design is a notable limitation, as while it offers insights into the antimicrobial properties of Ch-PMMA, it may not fully replicate the complexity of the human oral cavity, where factors like saliva flow, pH fluctuations, and diverse microorganisms could affect clinical efficacy. Additionally, the short-term nature of the study limits its conclusions, as long-term evaluations are necessary to assess the durability of the antimicrobial properties, which could diminish over time due to wear and chemical changes. The study also focused only on *C. albicans* and *S. mutans*, whereas the oral microbiome is far more diverse, and other pathogens may respond differently to Ch-PMMA. Furthermore, variability in chitosan distribution within the PMMA matrix, which was not accounted for, could lead to inconsistent antimicrobial effects across denture surfaces. The mechanical properties of the modified dentures, such as flexural strength and impact resistance, were also not extensively evaluated, though they are crucial for denture functionality and longevity. Lastly, while chitosan is generally biocompatible, the study did not thoroughly investigate potential allergic reactions or other adverse effects, emphasizing the need for comprehensive biocompatibility testing before clinical use. 

## Conclusions

The results of this study demonstrate that Ch-PMMA dentures exhibit superior antimicrobial efficacy compared to conventional heat-polymerized PMMA dentures. As the concentration of chitosan in the PMMA matrix increases, a significant reduction in microbial growth was observed against *C. albicans* and *S. mutans*. This enhanced antimicrobial property is attributed to the intrinsic antimicrobial nature of chitosan, which disrupts microbial cell walls and inhibits biofilm formation, thereby improving the overall hygiene and longevity of the denture material.

Clinically, the incorporation of chitosan into PMMA dentures offers several advantages for patients. Enhanced antimicrobial efficacy can lead to a reduction in oral infections such as denture stomatitis. This can result in improved oral health, greater comfort, and increased patient satisfaction. Additionally, the potential for fewer denture-related complications could reduce the need for frequent dental visits and adjustments, ultimately improving the quality of life for patients. The development of such materials aligns with the ongoing advancements in dental prosthetics aimed at enhancing patient outcomes through innovative material science. Successful future prospects could pave the way for widespread clinical adoption of this enhanced denture material, setting a new standard in denture fabrication and patient care.
